# American Academy of Dental Science

**Published:** 1895-10

**Authors:** 

**Affiliations:** American Academy Dental Science


### AMERICAN ACADEMY OF DENTAL SCIENCE.

The regular monthly meeting of the American Academy of Dental Science was held at Young's Hotel, Boston, January 2, 1895, President Smith in the chair. The paper for the evening was read by Dr. James H. Daly, of Boston; subject: "The Thorough Removal of Deposits in the Successful Treatment of Pyorrhœa."

(For Dr. Daly's paper, see page 605.)

DISCUSSION.

*Dr. Andrews.*—There is one kind of nodule that may be taken for nodular tartar. It is a deposit on the tooth-root very nearly like the deposit we find in forming exostosis, and I question whether it is not directly from the blood. Perhaps I can explain by saying that calcoglobulin in the periosteum in the form of minute globular bodies is given off from the blood-vessels, and these globules are deposited in a gelatinous substance where the globules merge together. Another stage in the calcifying process hardens the mass, and a bony deposit is formed. Now, I believe that some such process takes place in the formation of a certain kind of so-called nodular tartar,—that is, I believe these calco-spherites are deposited from the periosteal tissue on the surface of the tooth at a point near the end of the root, and it is true ossification of tissue,—not really a deposit of tartar, but a formation of bone. I have been studying the subject somewhat, and I have been surprised to see the number of these little nodules, so hard that they resemble the cementum rather than a tartar which you can scale off with an instrument. I must confess that I have as little success in treating this trouble as any I have to deal with. I think I modify the trouble, but it is seldom that I can see a certain cure. I have made a great many mouths very much better; but in the course of time they usually relapse and come for more treatment. To me it is the most puzzling of all the diseases we have to treat, and I am willing to state that I succeed less satisfactorily in this disease than in any other. I acknowledge that I do not understand it, and yet I have read almost everything that has been written upon the subject. I hope we shall get more light and information; something that will enable us to secure more definite results. I hear men make the statement that they cure this disease, but I am sorry to say that I do not have very great success in treating it.

*Dr. Daly.*—I do not like the idea of taking in everything that comes under the term of "pyorrhœa;" my few thoughts are not on pyorrhœa; one could write a volume about that, and I did not intend, when writing this paper, to include the whole subject. It was written simply with regard to instrumentation as an adjunct in the cure of pyorrhœa. The text-books all say "the deposits should be thoroughly removed." Well, there is a great deal in that, but the question is, how to thoroughly remove? The student in reading the text-book does not get much information, though sometimes, perhaps, it is as much as the author can give in the allotted space. I am confident that it is not an easy matter, especially with a large instrument, to remove this calcific matter, whatever it may be, and yet, in my opinion, upon this removal depends your ultimate success in the cure of the disease. I know that this is also Dr. Robinson's belief, and that he treats pyorrhœa with this thought in mind. In a case of alveolar abscess this calcific matter is often deposited at the apex of the root, and the filling of the root-canal does not cure the abscess. The pus continues to flow, (and that's all pyorrhœa is,—simply a flow of pus), and you may use carbolic acid and all sorts of things; but you will not be able to stop the pus until the tartar at the end of the root is removed, and that can only be done by instrumentation, and it takes a very fine, delicate instrument and a delicate touch to do it.

*Dr. Andrews.*—If a deposit was of the character that I mentioned,—what I believe to be from the calcoglobulin of the blood,—it would be almost impossible to remove it with an instrument. They can be removed, no doubt, but it would be as hard work as to remove cementum. You would be surprised to see some of the specimens I have under the microscope; they look like little elevations of bone on the surface of the root.

*President Smith.*—It would be interesting to see some of the instruments which members use for the removal of deposits.

*Dr. Daly.*—Here are just a few, and they are what I prefer. There is a difference of opinion in regard to keeping instruments sharp. Some instructors recommend having them dull,—not dull exactly, but with a bevelled edge; not a sharp cutting edge like this.

*Dr. Bradley.*—Perhaps the term "right-angle edge" would express the sharpening that Dr. Daly refers to. It seems to me, in the treatment of pyorrhœa, we often do not observe satisfactory methods. For instance, we may have a pocket around one tooth where there is a marked case of pyorrhœa and the pus is exuding. We take off the tartar from that tooth, then go on working around other healthy teeth with the same instruments which we used around the diseased tooth. It is possible to convey the disease to the teeth that are not so badly affected. Therefore, we should be very careful to wash our instruments before using them on the teeth which appear to be the least affected.

*Dr. Daly.*—Speaking of that, I have in my mind a case now where the right superior central, right lower cuspid and first bicuspid, and left inferior cuspid were the only teeth that were suffering from pyorrhœa in the mouth, and my thought was that one should be careful in working about the other teeth, as there is a possibility that the disease could be transferred. Else why should it exist in those teeth and not affect the mouth generally?

*Dr. G. T. Baker.*—I am much pleased with the thoughts of the essayist, and also with the instruments that have been passed around. The only improvement that I would suggest on the instruments is that, instead of being square, they be shaped more like this glass,—that is, made with a curved surface so as to conform to the tooth. As a help to the instrument, I have lately used sulphuric acid, and I find that it dissolves all the small particles and allows them to be removed much more easily. And besides that, it acts as an astringent on the gums, and causes them to adhere to the tooth, and is a benefit in that way. I use the pure sulphuric acid, not the aromatic, making about a three-per-cent. solution, which is about as strong as a person cares to take in the mouth; and I follow it with an alkali, either soda or magnesium, to counteract the effects of the acid. The instruments that Dr. Daly has shown are very good, and one can readily see how well adapted they are to the removal of the deposits about the anterior teeth; but what I want to see is an instrument that reaches down to the apex of the root on the distal surfaces of the molars, and enables us to get at the deposits that now seem quite impossible to reach.

*Dr. Belyea.*—When I first commenced practice, I had the pleasure of being associated for four months as assistant to Dr. Daly, and naturally I have used many of his methods. It was my custom, he will remember, to use peroxide of hydrogen not only in the ordinary fashion of applying it about the teeth, but also as a mouth-wash. Dr. Daly, being more conservative, perhaps, than I was, made some objections to the use of it as a mouth-wash; but I kept on, and have been using it right along, and have always been thoroughly satisfied with the result. Of course I believe in the ordinary use of the instruments, but I believe I cure more and help more than I otherwise would by the free use of peroxide of hydrogen; and I have seen no bad results from it. I do not believe that listerine does a particle of good, but rather does harm; while the peroxide not only acts as a tonic and a germ-killer, but also dissolves the tartar and makes its removal a great deal easier. I have been using for some time the Oakland Chemical Company's preparation; I like it better than Marchand's.

*Dr. Daly.*—Dr. Belyea said I was a little more conservative than he was, but it was simply conservatism; I always like to be on the safe side. You will notice there is sometimes quite a difference of opinion as to what it is safe to use. For instance, Dr. Baker uses a three-per-cent. solution of sulphuric acid, which he says he thinks is as strong as should be used in the mouth; now Dr. James Truman, of Philadelphia, uses a twenty-five-per-cent. solution of the chemically pure sulphuric acid, which is a pretty high solution. As we seem, by common consent, to have extended the discussion to include the entire treatment of pyorrhœa, perhaps it would be interesting to outline Dr. Truman's treatment as described by himself. In the first place, his treatment is to wash out the mouth with a solution of bichloride of mercury, one to two thousand; then, by instrumentation, to remove as thoroughly as possible all deposits; then bathe the roots with a twenty-per-cent. solution of sulphuric acid, and neutralize that by the use of bicarbonate of soda; then he packs sulphate of quinia thoroughly about the roots of the teeth. That treatment is also my treatment, with the one exception that I use aromatic sulphuric acid instead of chemically pure. The solution used by Dr. Truman seems pretty strong, but when you use aromatic sulphuric acid you are using twenty per cent. of the drug itself; it might be called twenty-per-cent. C. P.

*Dr. Bradley.*—May I ask if the essayist ever uses any agent to prevent pain? My own practice is to bathe the gums, before commencing to use the instruments, with cocaine; this modifies the pain of the operation considerably. I then follow with a preparation of peroxide of hydrogen, three per cent., and bichloride of mercury, one to one thousand, and generally prescribe this as a mouth-wash for the patients to use themselves. I have prescribed listerine, though less ready to do so now than formerly. I have advised the use of it in this manner: Patients, after brushing their teeth thoroughly, are to rinse out the brush and go over the teeth again. I do it to get the effect of the listerine and to enforce a more thorough cleansing of the teeth. There is one objection to the use of bichloride of mercury and peroxide of hydrogen as a mouth-wash: if it be continued long by the patients it will stain the teeth; this effect may be guarded against by the patient, as the stain can be removed without much difficulty by the application of a powder.

*Dr. Briggs.*—This is a very interesting subject, and it is hard to approach it without going into the whole field of pyorrhœa alveolaris, its cause, prevention, treatment, and cure. I think Dr. Daly has struck the key-note when he refers to the importance of thorough use of instruments in the treatment of this disease. When I first entered into practice, the only attempt at treatment was to scale the teeth in the ordinary manner; and my first instinct was to wander into the fields of medicine and apply something which would help to restore the gums to a healthy condition, thinking to arrest the disease in that way. Now, while I still continue to apply the medicines, I believe the foundation of the treatment is to remove the tartar with the instruments; and I do not believe—at least I have not seen it in any case in my experience, which has covered about seventeen years—that we have this disease without deposits of calcareous matter; and the cases that have been reported of pus exuding from the gums where this deposit was not present, I am not inclined to accept, because I think the observers did not explore carefully and delicately enough to find the deposit. I believe if we see the case before the disease has progressed too far that we can make cures; if it is allowed to exist too long, it extends to the apex of the root and destroys the alveolar process, and there is no hope of saving the tooth. I have found, as Dr. Andrews has described, some deposits that simulate exostosis, and I would like to cite a case. About six weeks ago I saw a patient whom I had been treating for this trouble, and a year ago I thought I had the case under pretty good control. The patient went abroad and returned a couple of months ago and came to me with the tooth in a bad condition. I treated it to the best of my ability, but apparently without doing a particle of good, and I finally took it out. At the apex of the root, beyond where my instrument could go, I found a hard, smooth deposit. I removed this, and the tooth being in good condition, I drilled the socket a little deeper—say one sixteenth of an inch—and put the tooth back, holding it with a splint. Last Saturday the splint was removed, and I am in hopes that I will succeed in getting further service from this tooth. This deposit was entirely beyond the reach of any instrument; still I do not believe that it came there except through an original avenue, or socket or pocket, which began at the margin of the gum; and if one can get at these pockets soon enough, before they have extended very far up the root, there is no reason why one should ever lose a tooth from this disease. I might say that I use in my practice, in connection with the instruments, trichloracetic acid, and I find it helps to remove the calcareous matter; and as for the instruments, I do not think the inventive mind of the dentist can be employed too fast or too assiduously in devising these points. We have reached the limit of the instruments which we now have, and are not able to obtain the results which we desire with them. Some of you are much more inventive than others, and there is at present nothing in the whole field of dentistry where ingenuity can be employed to better advantage than in the improvement of our instruments, that we may be enabled to reach the parts which it is necessary that we should, but which are at present without the limits of our instruments. I would like briefly to go over the treatment that I am at present using. First, wipe the gums with a twenty-per-cent. solution of cocaine, to numb them as far as possible; then cleanse the mouth with pyrozone; then, while I am getting my instruments ready, I instruct the patient to keep rinsing the mouth with the solution made by dissolving one of Seiler's tablets in a glass of water; I then wipe around carefully in the pockets with trichloracetic acid, which I have found to be the best thing I have ever used for the softening of the tartar and the dissolution of those little fragments that cling to the root after you have removed the larger portions; after this I proceed with the instruments, and I consider this the most important part of the whole treatment. I have scaled teeth until I would be sure that they were absolutely clean, and then look at them in a week and would find a number of those little dark spots, which would necessitate going over the whole field again. It is easy to be deceived in this work; for, as Dr. Andrews says, some of these spots are so smooth and hard that the instrument glides over them, and you would be sure that you had removed all the calcareous matter on the root; but as long as any little spot remains you will continue to have your pocket, and the case will never be cured. Careful work, then, with the instrument is the most important thing. After that is thoroughly done—mark you, there are many cases where, with our present instruments, it is impossible for us to remove all the deposits, and such are for the present beyond our relief—you will have no difficulty in saying that you have seen cases that have been cured. After you have removed the tartar, then, of course, comes the treatment of the pocket; and it suggests itself to you to make that aseptic; to remove all the dead tissue, which I burn off with caustic potash, and then close up the pocket by applying an astringent; and the astringent that I use is the chloride of aluminum. Of course, you can use any other astringent that you are accustomed to; but I have had such uniform success with chloride of aluminum that I am glad to recommend it. There was one other point that Dr. Banfield particularly spoke of,—that of dead pulps. There are a great many cases where the disease has gone up the root some distance,—not apparently very seriously; and yet, it has been my experience, you will find that it has affected the pulp of the tooth, and you have a complication of a dead tooth with the other mischief; and you may go on, treating the pyorrhœa indefinitely, without affecting a cure, simply because the dead pulp needs to be taken out. Other cases come to your notice before the pulp dies; and if you find with a bad case of pyorrhœa that there is any indication of pulpitis, it seems to me much better to take the "bull by the horns" and remove the pulp, which simplifies the treatment very much.

*President Smith.*—I would like to ask Dr. Briggs, in connection with this treatment, whether he now uses (I think I am right in assuming that he formerly used) sponge-grafting for the pockets—packing in sterilized sponges for the tissues to grow about?

*Dr. Briggs.*—I still use the sterilized sponge a great deal; I would not be without it in my practice. It is not so applicable in these cases as I would like, because one side of the pocket is bone, and it is very hard, in that condition, to get the proper grafting into the sponge; and I find if you have a bad pocket, the best thing you can hope for is to cure and then endeavor to make the gum adhere to the tooth as best you can; you are sure to have a certain percentage of retraction of the gum about the teeth, any way. If the gum is healthy, you can, by the use of astringents, usually get a satisfactory result. If I may be allowed to digress a little, I would like to refer to the value of sponge-grafting for other things. I wish you all would use it for curing old fistulæ and as a sponge-tent for opening into an abscess; for instance, if you have opened into an abscess or down to the apex of a root that is troublesome, and succeeded in finding the cavity or track of inflammation and pus, after cleaning it out as best you can, pack in sterilized sponge, which acts as a perfect drainage-tube for any further formation of pus and prevents other material from getting in there; and when the tissues begin to heal the sponge becomes absorbed, as you know, like a piece of catgut. In a great many places where the inflammation has left cavernous spaces about the roots of the tooth, I pack the sponge in there, and it can be left for some time without becoming at all offensive.

*President Smith.*—The reason I spoke about it was because I had tried it in one or two cases and was not so successful as I had hoped to be. There is one thing that I have not done in the past as much as I shall do in the future,—that is, after removing the deposits about the roots of the first and second molars, where the disease is advanced, you find these great, deep pockets where everything lodges. I am in the habit now of giving to my patients a solution of carbolic acid together with a syringe, with instructions to keep the pockets thoroughly clean. These pockets serve as a catch-all for the foods and secretions and greatly retards the return of the tissue to normal use; when this precautionary measure is used, the improvement is more noticeable.

*Dr. Briggs.*—I would like to speak of one more thing, which I think will be found to be very useful in our treatment of pyorrhœa in the future. In our improvements in local anæsthesia there are great strides being made, as you have noticed, in finding out what drugs can be used with absolute safety; and there is a most interesting paper in the last number of the Journal of the American Medical Association describing Schleich's method and giving the formula of an absolutely safe preparation to be used as a local anæsthetic. If this method is completely successful, I believe that we will be able to open the gums a little around the tooth, and then, packing in the sponge, force up the gum to the tooth. I have no doubt that many cases can be closed in that manner.

William H. Potter, D.M.D.,
*Editor American Academy Dental Science.*

